# Chitosan hydrogel for topical delivery of ebastine loaded solid lipid nanoparticles for alleviation of allergic contact dermatitis†

**DOI:** 10.1039/d1ra06283b

**Published:** 2021-11-22

**Authors:** Tasbiha Kazim, Abeer Tariq, Muhammad Usman, Muhammad Faisal Ayoob, Ahmad Khan

**Affiliations:** Department of Pharmacy, Quaid-i-Azam University Islamabad Pakistan akhan@qau.edu.pk +92 336158004; National Veterinary Laboratory Islamabad Pakistan

## Abstract

Ebastine, is an antihistamine drug that exerts its effect upon oral administration in humans for the treatment of allergic contact dermatitis (ACD), it also has some systemic side effects like gastric distress, headache, drowsiness, and epistaxis. Moreover, topical corticosteroids are used for treatment of ACD, which causes the human skin to lose its thickness and elasticity. Hence, ebastine-loaded solid lipid nanoparticles (E-SLNs) were prepared and their topical efficacy against allergic contact dermatitis was determined. Compritol 888 ATO and tween 80 were used to prepare E-SLNs by cold dilution of the hot micro-emulsion. E-SLNs were optimized statistically by employing a central composite design using Design-Expert® version 11.0. Optimized E-SLNs showed spherical surface morphology, zeta potential of −15.6 ± 2.4 mV, PDI of 0.256 ± 0.03, and particle sizes of 155.2 ± 1.5 nm and th eentrapment efficiency of ebastine was more than 78%. Nanoparticles were characterized using FT-IR, XRD, and TEM. An E-SLNs loaded hydrogel was prepared using chitosan as a gelling agent and glutaraldehyde as a crosslinker. *In vitro* drug release studies performed for 24 hours on the E-SLNs dispersion and E-SLNs loaded hydrogel showed a sustained release of maximum 82.9% and 73.7% respectively. *In vivo* studies were conducted on BALB/c mice to evaluate the topical efficacy of the E-SLNs loaded hydrogel for allergic contact dermatitis. ACD was induced on the ear using picryl chloride solution. After induction, ears were treated daily with the E-SLNs loaded hydrogel for 15 days. Swelling behavior, mast cell count, and histopathological studies of the ear confirmed that the hydrogel alleviated the symptoms of allergic contact dermatitis.

## Abstract 2

Ebastine exerts its effect upon oral administration in humans for the treatment of allergic contact dermatitis (ACD), but it has some systemic side effects. Hence, ebastine-SLNs loaded hydrogel was prepared to increase topical efficacy of ebastine.

## Graphical abstract

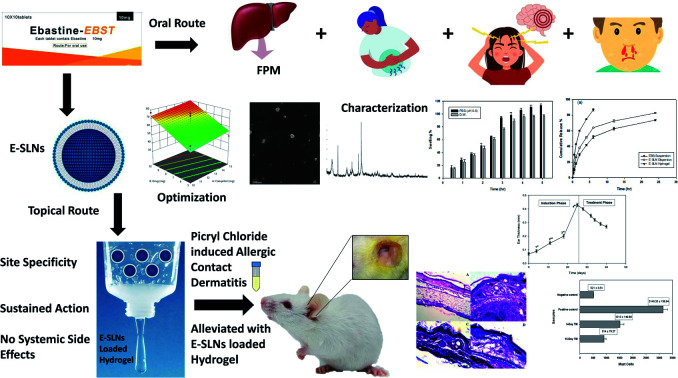

## 1. Introduction

Allergic contact dermatitis (ACD) is a type-IV or delayed type hypersensitivity reaction triggered by a tiny molecule (less than 500 Daltons) that comes across a sensitive individual's skin.^1^ When a foreign material encounters the skin and forms an antigen complex with hapten protein, sensitization occurs.^2^ After first exposure to the allergen, the induction phase comprises various events and is completed when a person is sensitized and able to provide a positive ACD response.^3^ After elicitation, the effector phase commences and leads to clinical manifestations of ACD. It takes at least three days to many weeks to complete the process of the induction stage, whereas the effector phase reaction takes 1–2 days.^4^ The sensitized T cells trigger an inflammatory cascade when the epidermis is re-exposed to the antigen, resulting in skin changes correlated with allergic contact dermatitis. Allergic contact dermatitis is a frequent source of occupational skin illness, accounting for around 20% of all health problems at work.^5^ Poison ivy is a common cause of ACD, and it appears as linear stripes on the skin where the plant interacts.^6^ Nickel is another prevalent cause of ACD because nickel-containing necklaces and earrings are commonly worn. Rubber gloves are a leading cause of persistent dermatitis. Additional agents include hair colours, preservatives, fabrics, sunscreen, perfumes, and allergies.^5^

ACD appears to be the outcome of both genetic trends and environmental factors in some of the groups at increased risk. Genealogy showed that ACD developed at higher rate, suggesting a hereditary susceptibility; however, the shared environment was a confounding factor. Women appear to be more prone to getting ACD. This disparity is due to exposures rather than sex differences; women have a greater incidence of nickel allergy, possibly due to the increased rate with which they wear jewellery.^7^ ACD is not uncommon in children and can cause severe clinical problems. ACD accounts for up to 20% of all childhood dermatitis. When compared to adults, ACD is uncommon in young children, and it develops more commonly as children get older and are exposed to more environmental allergens.^8^ Histamine is the key mediator in the production of allergic symptoms that is produced by the body and oral H1-antihistamines are one of the highly prescribed therapies for ACD. Ebastine is an oxypiperidine-based second-generation antagonist of H1-histamine receptor. Ebastine has fast absorption time and lengthy duration of action, at least partially mediated by the production of a more potent acid metabolite *i.e.*, carebastine.^9^ Its antagonizing activity frequently prevents histamine activation, particularly in cases of acute hypersensitivity.^10^ Patients with allergic dermatitis, cold urticaria, demographic urticaria, mosquito bites, atopic asthma, and the common cold have shown positive results in some studies. Ebastine is usually administered *via* the oral route, however, it has its side effects.^11^ The most prevalent adverse events were headache 7.9%, sleepiness 3%, and mouth dryness 2.1%. Abdominal pain, epistaxis, dyspepsia, rhinitis, nausea, sinusitis, and sleeplessness were among the 1% of adverse events.^12^

Most medications pose a major challenge to the formulation researchers due to their poor water solubility, that is also superintend for low bioavailability.^13^ The encapsulation of the drug in a particulate carrier system is one way to solve the problem. Due to its unique capacity, solid lipid enhances the bioavailability of medicines with poor water solubility, it has emerged as a very appealing alternative among other carriers for drug delivery. SLNs are attractive as innovative colloidal drug carriers for topical usage because they provide a good mechanism for delivering pharmaceuticals *via* multiple application routes.^14^ The benefits of these carriers include minimal skin irritation, regulated release, and active substance protection. SLNs appear to be ideally suited for usage on injured and inflamed skin since they are constituted of non-toxic and non-irritative lipids.^15^ Furthermore, SLNs exhibit specific occlusive qualities due to the development of an integral film over the skin surface after drying, which reduces *trans*-epidermal water loss and promotes drug penetration through stratum corneum.^16^ Nano-sized SLNs, in addition to having a highly selective surface area, aid in the adhesion of the encapsulated medicine with the stratum corneum. Solid lipid nanoparticles can establish close contact with corneocyte cluster superficial connections and the furrows between corneocyte, which may encourage aggregation for several hours, allowing for prolonged drug release. SLNs also have a high pharmacological payload and can incorporate lipophilic and hydrophilic medicines.^15^

Administration of ebastine through oral route compromise its therapeutic efficacy through degradation to carebastine due to first-pass metabolism, low solubility that causes low plasma concentration, and have many systemic side effects. Oral ebastine is used with topical corticosteroids for the treatment of contact dermatitis. But topical steroid causes skin atrophy and skin loses its elasticity and thinning could result in skin striae.^17^ To surmount these concerns, ebastine was encapsulated into solid lipid nanoparticles, to enhance its solubility and to incorporate them into chitosan hydrogel for topical delivery. Chitosan was selected due to its anti-inflammatory property.^18^ These components are most appropriate because they are biodegradable, biocompatible, and non-irritant. Ebastine-loaded solid lipid nanoparticles will be delivered topically through hydrogel on the site of contact dermatitis. Ebastine will act as a mast cell stabilizer and alleviate allergic symptoms.^19^ Ebastine released in a sustained pattern will provide a prolonged effect after a single application of E-SLNs loaded hydrogel.

## 2. Material and methods

### 2.1. Materials

Ebastine was obtained from the Global pharmaceuticals. Compritol 888 ATO (Glyceryl Behenate) was received as a gift from Morgan Chemicals (Gattefosse, Germany), glutaraldehyde 70% (Sigma-Aldrich Chemie). Chitosan was purchased from Sigma Aldrich, M.W. = 310–375 kDa (CAS-9012-76-4). Picryl Chloride was obtained from Sigma Aldrich. Tween-80 was gifted by Horizon Pharmaceuticals. Olive oil (pharmaceutical grade) was obtained from Scotmann pharmaceuticals.

### 2.2. Animals

Eight to ten weeks old BALB/c male mice were purchased from the National Institute of Health, Islamabad, Pakistan, for animal studies. Mice were transported to National Veterinary Laboratory, Islamabad to conduct the studies. They were held in polycarbonate cages, and for the whole study period. Standard laboratory pellets and tap water (*ad libitum*) were fed. The animal house was air-conditioned with 14 hours light and a dark 10 hours cycle (temperature 23 ± 2 °C and relative humidity 55 ± 5%). Procedures for conducting animal research were implemented in accordance with the NIH Policy Animal Welfare Act with the consent of the Quaid-i-Azam University BIO Ethics Committee, with assigned protocol no. BEC-FBS-QAU2021-310.

### 2.3. Preparation of ebastine loaded solid lipid nanoparticles

**Fig. 1:**
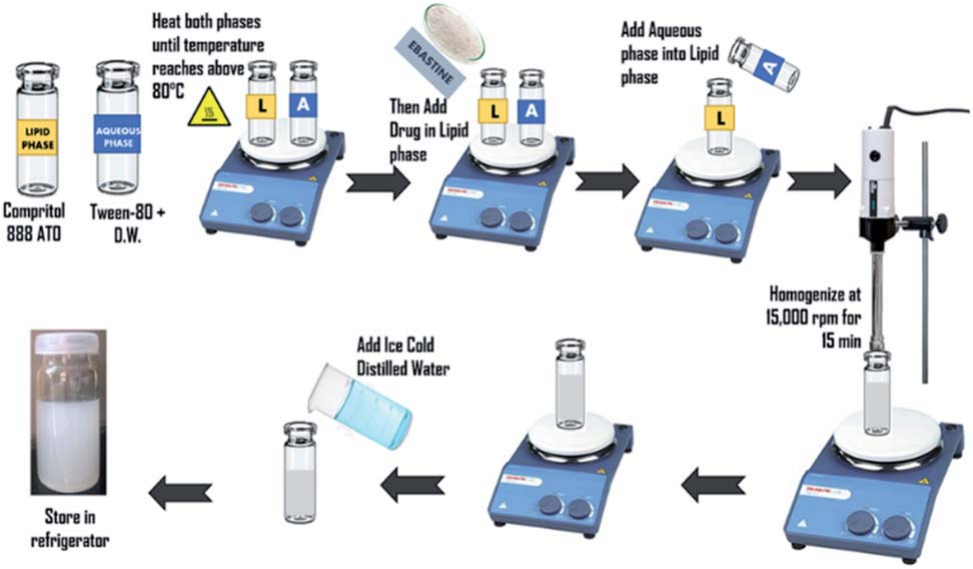
Preparation of ebastine loaded solid lipid nanoparticles.

Nanoparticles were prepared by the hot homogenization method following cold dilution of micro-emulsion. To obtain ebastine-loaded solid lipid nanoparticles (E-SLNs), tween-80 was dissolved in 5 mL of distilled water using a magnetic stirrer. Compritol 888 ATO and ebastine were weighed. Compritol was heated up to 80 °C on a hot plate to melt it, referred as the lipid phase. Tween-80 with distilled water (aqueous phase) was heated to the very same temperature as the lipid phase.^20^ Ebastine was added into the melted lipid and then the aqueous phase was added. Homogenized at 15 000 rpm for 15 minutes to form the initial w/o emulsion while maintaining the temperature at 75 °C.^21^ Obtained micro-emulsion was diluted with ice-cold distilled water which resulted in the formation of nano-emulsion and the subsequent formation of ebastine loaded solid lipid nanoparticles by lipid precipitation (Fig. 1).^22^

### 2.4. Experimental design

A two-factor four-level Central Composite Design (CCD) was employed for the optimization of Ebastine-loaded SLNs using Design-Expert® version 11.0. The influence of two independent variables *i.e.*, solid lipid amount (*X*_1_), Drug amount (*X*_2_) on four dependant variables/responses *viz.* particle size (*Y*_1_), polydispersity index (*Y*_2_), zeta potential (*Y*_3_) and entrapment efficiency% (*Y*_4_) were evaluated.^23^ Ten experimental runs with two centre points were generated as per CCD. Formulations were made corresponding to the runs generated by the software. The results of responses were concurrently fitted into different mathematical models *i.e.*, linear, quadratic, 2FI, and cubic models, and statistical significance was analysed. At a *p*-value less than 0.05, the model was regarded as significant.^24^

### 2.5. Preparation of E-SLNs loaded hydrogel

**Fig. 2:**
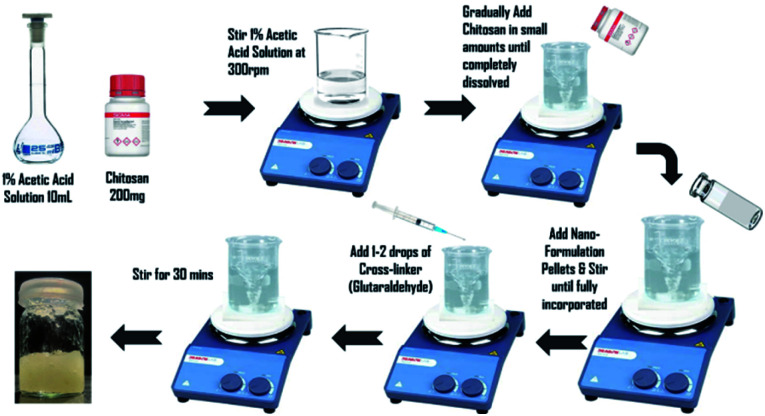
Preparation of E-SLNs loaded hydrogel.

To prepare 2% w/v chitosan gel, 200 mg of chitosan powder were weighed and then dissolved in 10 mL of 1% v/v acetic acid. Stirred mechanically at room temperature. Pellets of E-SLNs formulation were added and mixed homogeneously. Then 1–2 drops of 0.1 M glutaraldehyde were added and stirred for 30 minutes to increase cross-linking reaction time. The resulting crosslinked chitosan hydrogel was poured into a closed container and stored at room temperature (Fig. 2).^25^

### 2.6. Characterization of E-SLNs

#### 2.6.1. Particle size, PDI, and zeta potential

Particle size, PDI, and zeta potential of nanoparticles were measured in a cuvette using Malvern zeta sizer Vσ.7.12. E-SLNs were diluted with deionized water (1 : 10) before analysis.

#### 2.6.2. Entrapment efficiency and drug loading capacity%

The entrapment efficiency and drug loading capacity% were determined by first separating the nanoparticles from the aqueous medium by ultra-centrifugation. The amount of unentrapped drug in the supernatant was quantified spectrophotometrically at a wavelength of 253 nm by diluting supernatant in methanol with a ratio of 1 : 10. *R*^2^ value and slope equation of calibration curve was used. Entrapment efficiency% and drug loading capacity% was determined by the following equations:
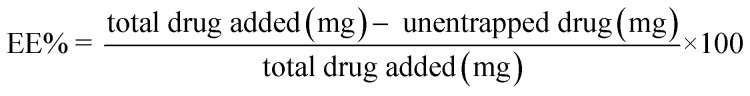

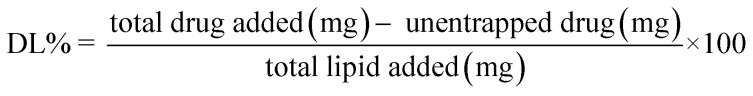


#### 2.6.3. Transmission electron microscopy (TEM)

For TEM evaluation, a drop of diluted E-SLNs was spread on a membrane coated copper grid. Within a minute, the surplus fluid was removed, and the surface of grid was dried at ambient temperature. Consequently, the stained grid was analysed under TEM.

#### 2.6.4. Fourier transform Infrared spectroscopy (FT-IR)

Fourier Transform Infrared (FT-IR) analyses were performed for physicochemical characterization on ebastine, compritol, physical mixture, and ebastine loaded solid lipid nanoparticles. The samples were freeze-dried and mixed homogenously with potassium bromide, and then the blend was compressed into discs using a hydraulic compressor by applying a pressure of almost 10 tons in 2 minutes. The discs were placed in an infra-red-light pass of FTIR spectroscope. The IR spectrums were recorded in a region of 400–4000 cm^−1^.

#### 2.6.5. Powder X-ray diffractometry (PXRD)

XRD of ebastine, compritol, physical mixture, and lyophilized optimized E-SLNs was performed for identification of phase. It was performed on an XPERTPRO diffractometer, between 2*θ* angles around the range of 0–70°. Copper was sourced as an anode material; alpha and beta radiations were collected at 40 mA tube current and 45 kV tube voltage. Measurements were taken at a temperature of 25 °C.

### 2.7. Characterization of E-SLNs loaded hydrogel

#### 2.7.1. Visual appearance and pH

The physical appearance, grittiness, and homogeneity of the chitosan hydrogel were evaluated by visual observations.^26^ To determine pH, 1 g of chitosan hydrogel gel was weighed and dissolved into 20 mL of distilled water and the solution was stored for 2 hours. The pH of the hydrogel was measured by placing the electrode of the pH-meter in contact with the gel and was allowed to equilibrate for 1 minute before measurement.^27^

#### 2.7.2. Spreadability%

Gel spreadability was evaluated by putting 0.5 g of hydrogel inside a 2 cm (*θ*) circle drawn on a glass slide. A second glass slide was placed on the first glass slide and for 5 minutes, 500 mg of weight was placed on the upper glass slide, and the diameter of the gel after spreading (*A*_2_) was measured.^28^ Spreadability% was determined by the following equation:
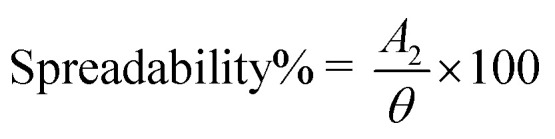


#### 2.7.3. Drug content determination

To evaluate the uniform distribution of ebastine in the hydrogel, a drug content analysis was performed. 1 g of hydrogel was dissolved in 100 mL of methanol to lyse the vesicles. The solution was sonicated and then filtered *via* a 0.45 μm membrane filter. 5 mL of filtrate was diluted to 50 mL in methanol. Absorbance of the solution was measured at 253 nm using UV-spectrophotometer.^28^ Drug content was determined by the following formula:
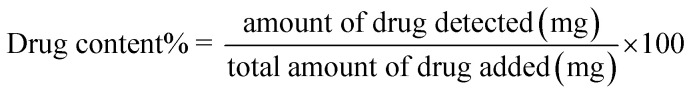


#### 2.7.4. Gel swelling

Gravimetric method was used to measure the swelling index of chitosan hydrogel in PBS 5.5 and water. A weighted amount of gel was placed in PBS 5.5 and water in a Petri dish. The swollen hydrogels were separated from the medium and weighed at precise time intervals of 0, 0.5, 1, 1.5, 2, 2.5, 3, 3.5, 4, 4.5, and 5 hours until the weight of the swollen hydrogel remained consistent. Extra water was removed from the hydrogel surface by lightly tapping it with a filter paper.^29^ Swelling indices were measured using the following formula:
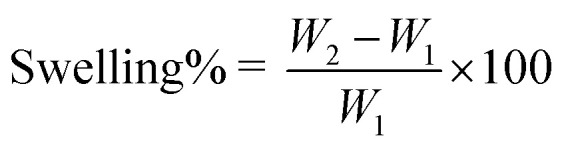
where *W*_1_ is the initial weight of hydrogel and *W*_2_ is the weight of gel after swelling.

#### 2.7.5. Rheological behaviour

The rheological behaviour of E-SLNs loaded hydrogel was performed on viscometer (LVDV-I Prime Brookfield Viscometer, USA) using spindle-64. For each sample at different shear rates from 2 to 100 s^−1^ and the resulting viscosity was evaluated in centipoise.^30^

#### 2.7.6. Extrudability

Extrudability was determined by pressing hard on the crimped end of a closed collapsible aluminium tube containing hydrogel. Once the cap was removed, the hydrogel extruded until the pressure was relieved. The weight needed to extrude a 0.5 cm ribbon of the hydrogel in 10 seconds was measured. The average extrusion pressure was recorded in grams.^26^

### 2.8. *In vitro* release studies

The *in vitro* drug release of ebastine from ebastine suspension, E-SLNs, and E-SLNs loaded hydrogel was evaluated by dialysis bag method at pH 5.5 and 7.4 for 24 hours. Suspension of ebastine was prepared in release medium. After ultracentrifugation, a specific quantity of ebastine-loaded nanoparticles was redispersed in a little amount of phosphate buffers (pH 5.5 and 7.4) for nanoparticles and incorporation in hydrogel.^31^ Afterwards the suspension, nanoparticles, and hydrogel were transferred to a dialysis bag, tied at both ends with thread, and immersed in beakers containing buffers. The beakers were placed in a bath shaker and the temperature was kept at 37 ± 1 °C.^32^ At pre-defined time intervals, 2 mL of samples were withdrawn, and an equal amount of freshly prepared release medium was replaced to preserve sink conditions. Samples were analysed spectrophotometrically at 253 nm to measure drug release. Various kinetic models were applied to determine the release mechanism of ebastine from each formulation using DDSolver.^33^

### 2.9. *Ex vivo* permeation studies

#### 2.9.1. Preparation of skin

The skin of BALB/c mice was used for permeation studies. Hairs were removed from abdominal skin through shaving. Mice were euthanized and abdominal skin was removed surgically. The integrity of the skin was checked through visual inspection. The skin was cleaned using phosphate buffer pH 7.4. Fat tissues were removed precisely to avoid skin damage. Skin was placed in PBS 7.4 before use in permeation studies.^34^

#### 2.9.2. Drug permeation

The skins were placed on Franz diffusion cells with the surface area of 0.77 cm^2^ and 5 mL volume was placed in the receiver compartment. The dermal surface of the skin was subjected to the receptor fluid containing Methanolic PBS (pH 7.4). The stratum corneum side was kept in contact with donor compartment content. 0.5 g of E-SLNs hydrogel and ebastine hydrogel were placed in each donor compartment, to uniformly cover the whole surface of the skin. The temperature of the cell was kept at 37 ± 1 °C. Samples were taken from the receptor compartment through a sampling port at a specific time of 0.25, 0.5, 1, 2, 4, 6, 12, and 24 hours. Samples were evaluated using a UV-spectrophotometer at the wavelength of 253 nm.^35^

### 2.10. Stability studies of E-SLNs loaded hydrogel

According to ICH guidelines, stability tests of the E-SLNs loaded hydrogel was carried out. A 6 month study was conducted at temperatures of 4 °C ± 2 °C and 25–35 °C with a relative humidity of 75 ± 5%, for 0, 1, 3, and 6 months. Drug content and pH were assessed. The E-SLNs loaded hydrogel was also examined physically for colour, grittiness, and phase separation.^29^

### 2.11. Skin irritation studies

BALB/c mice were used to evaluate the safety of E-SLNs hydrogel through a skin irritation study. Mice were divided into three groups (*n* = 3) and their hairs were shaved. Group I was negative control, group II was positive control (treated with 0.8% formalin), and group III was treated with E-SLNs hydrogel. After the application of formulations, mice skins were observed for edema and erythema for 24 hours. The Draize score method was used, primary dermal irritation (PDI) and primary dermal irritation index (PDII) were calculated. After 24 hours, mice were euthanized, and respective skins were removed surgically. Skins were fixed in 10% neutral buffered formalin (NBF). Haematoxylin and eosin staining was performed on the paraffin sections and observed under the microscope.^36^

### 2.12. *In vivo* allergic contact dermatitis studies

#### 2.12.1. Induction of ACD

Male BALB/c mice were distributed into 3 groups (*n* = 6). Group A was labelled as the negative control group, group B was positive control which only received picryl chloride sensitization and group C was treated by E-SLNs loaded hydrogel after sensitization. Group B and C were sensitized by applying 150 μL of 5% picryl chloride solution to the shaved abdominal skin. After sensitization, at 4^th^, 11^th^, 18^th^, and 25^th^ days right ear was topically sensitized with 20 μL of 0.8% picryl chloride solution. Acetone and olive oil with a ratio of 4 : 1 was applied to the left ear as vehicle in the same manner.

#### 2.12.2. Alleviation of ACD *via* application of E-SLNs hydrogel

After complete induction of contact dermatitis, the right ear of group C mice were topically treated with E-SLNs loaded hydrogel for 15 days once daily.^37^ The ear thickness of each mice treated with picryl chloride or vehicles was measured using a micrometre, 24 hours after the first, second, third, and fourth sensitization and hydrogel treatment.^38^ A graph was plotted between time in days and ear thickness (mm) to assess ear swelling behaviour. For histopathological examination, mice from each group were euthanized after complete induction and E-SLNs loaded hydrogel treatment. Ears were surgically removed and fixed in 10% normal buffered formalin. 2 μm paraffin sections were stained with toluidine blue and haematoxylin and eosin (H & E). In toluidine blue-stained sections, the number of mast cells was evaluated with an area of 30 to 40%, in toluidine-stained slides using microscopy and ImageJ software.^39^

### 2.13. Statistical analysis

All measurements were repeated three times. Results were presented as mean ± S.D. Statistical analysis was performed by one-way analysis of variance (ANOVA). Values of swelling percentage and mast cell count of positive control and treatment group were compared with negative/saline control group and the difference was assessed with Student's *t*-test (Excel Microsoft 365, USA). A *p*-value less than 0.05 was regarded significant.

## 3. Results

### 3.1. Optimization of E-SLNs

Independent variables, solid lipid concentration (*X*_1_), and drug concentration (*X*_2_) had a significant effect on response variables including particle size (*Y*_1_), polydispersity index (*Y*_2_), zeta potential (*Y*_3_), and entrapment efficiency (*Y*_4_) as per CCD. The particle size (PS), PDI, zeta potential (ZP) and EE% values for the ten runs showed a variation from 74.5 nm to 185 nm, 0.233 to 0.586, −6.16 to −16 mV, and 35.3% to 78.8% respectively (Table 1). Best fitted model for particle size according to CCD was 2F1. Significant model for PDI was quadratic. Both zeta potential and entrapment efficiency followed linear model. Equations generated for each response are as follows:PS (*Y*_1_) = 658.18290 − 40.89641 × *X*_1_ − 51.57331 × *X*_2_ + 3.988 × *X*_1_ × *X*_2_PDI (*Y*_2_) = 0.342882 × *X*_1_ − 0.051679 × *X*_2_ − 0.011480 × *X*_1_ × *X*_2_ − 0.011480 × *X*_1_^2^ + 0.011760 × *X*_2_^2^ − 1.52544ZP (*Y*_3_) = 6.38844 − 0.938793 × *X*_1_ − 0.606070 × *X*_2_EE% (*Y*_4_) = 9.04872 + 1.43990 × *X*_1_ + 4.92701 × *X*_2_

**Table 1:** Optimization of E-SLNs

Code	Factor 1 (*X*_1_)	Factor 2 (*X*_2_)	Surfactant	Response 1 (*Y*_1_)	Response 2 (*Y*_2_)	Response 3 (*Y*_3_)	Response 4 (*Y*_4_)
	A: Compritol (mg)	B: Drug (mg)	Tween-80 (mg)	P.S. (nm)	PDI	Z.P. (mV)	EE (%)
TKF1	16.0355	7.5	30	74.53 ± 2.4	0.233 ± 0.03	−9.24 ± 0.04	72 ± 3
TKF2	12.5	3.96447	30	135.2 ± 6.6	0.489 ± 0.07	−7.74 ± 0.86	35.3 ± 1
TKF3	12.5	7.5	30	137.1 ± 5.1	0.421 ± 0.05	−8.23 ± 2	73 ± 5.1
TKF4	8.96447	7.5	30	185 ± 8.2	0.258 ± 0.02	−6.16 ± 0.5	50.8 ± 3.4
TKF5	12.5	11.0355	30	101 ± 5	0.586 ± 0.04	−9.95 ± 1	73.3 ± 2.5
TKF6	10	10	30	137.1 ± 1.3	0.571 ± 0.07	−7.76 ± 0.7	76.8 ± 2
TKF7	15	5	30	98.4 ± 5.2	0.413 ± 0.01	−16 ± 0.2	49 ± 0.7
TKF8	12.5	7.5	30	137 ± 1.3	0.421 ± 0.03	−9.485 ± 0.4	73 ± 2
TKF9	10	5	30	180 ± 9.4	0.381 ± 0.06	−7.3 ± 0.33	55 ± 6.3
TKF10	15	10	30	155.2 ± 1.5	0.256 ± 0.03	−15.6 ± 2.4	78.8 ± 3

The synergistic effect is presented by a positive sign with the independent variable, while the antagonistic effect is presented by a negative sign. The *p*-values for PS, PDI, ZP, and EE% were 0.0208, 0.0134, 0.0473, and 0.0283 respectively, which shows the significance of each model. Observed *f*-values for PS, PDI, ZP, and EE% were 7.16, 13.27, 4.87, and 6.19 respectively, which implies that all fitted models are significant.

**Fig. 3:**
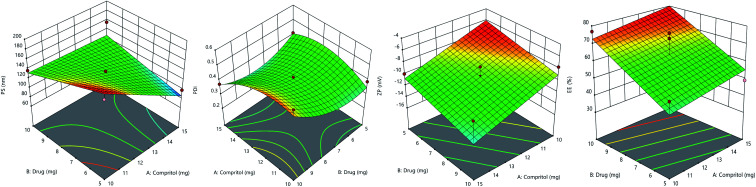
Response surface plots of particle size, poly dispersity index, zeta potential, and entrapment efficiency%.

3D response surface plot for particle size is shown in Fig. 3. The lipid and drug concentration have a positive effect on particle size, as presented in the 2F1 equation for particle size. It was noted that as the solid lipid content increased, the particle size increased as well, however, the change was non-significant. It is indeed possible that the non-significant increase in particle size is related to the lipid content's narrow range of 10 to 15 mg. Particle size was significantly affected by drug concentration. When the concentration of drug was increased from 5 to 10 mg, the particle size also increased.^40^

Fig. 3 shows a 3D response surface plot for PDI. The quadratic equation shows that lipid and drug concentration have a disparate effect on PDI. Initially, PDI increased with an increase in the concentration of drug and lipid, but afterward, it decreased with further increase. In case of drug concentration, PDI decreased initially but later it increased.

The 3D response surface plot of zeta potential is shown in Fig. 3. The linear equation of ZP shows that both factors have a negative impact on zeta potential. With an increase in drug and lipid concentration, ZP decreased towards less negative values, making formulation less stable.^41^

While linear equation of EE% showed that lipid and drug concentration have positive impact, increase in concentration of drug and lipid increased the drug entrapment. EE% is high due to presence of long fatty chain compritol and tween-80 in high concentration. Formulation TKF 10 was chosen as optimized formulation.

### 3.2. Characterization of E-SLNs

#### 3.2.1. Particle size, PDI and zeta potential

**Fig. 4:**
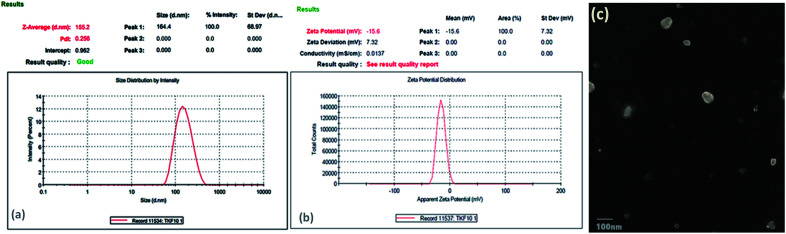
(a) Particle size distribution and PDI of optimized E-SLNs. (b) Zeta potential of optimized E-SLNs. (c) Transmission electron microscopy images of prepared optimized E-SLNs.

The particle size of E-SLNs was within nano-scale range, which is necessary for optimal penetration of E-SLNs into the skin. The particle size of TKF 10, the optimized E-SLNs was 155.2 nm with a PDI of 0.256 as shown in Fig. 4(a). Low value of PDI shows uniform size distribution in the formulation.

The zeta potential of optimized formulation was −16 mV, which validates sufficient stabilization to the formulation (Fig. 4(b)). The low value of ZP is due to the presence of tween-80 which is a high molecular weight surfactant.

#### 3.2.2. Entrapment efficiency and drug loading capacity%

The entrapment efficiency% of optimized E-loaded SLNs was 78.8 ± 3%, which is adequate and could be credited to the high solubility of ebastine in the compritol. Optimized formulation showed a% DL of 36.94 ± 4.2%.

#### 3.2.3. Transmission electron microscopy (TEM)

Transmission electron microscopy was used to investigate the morphology of E-SLNs. Particles in the nanometre range can be seen in the TEM images. It was determined that the particle size was approximately 100–150 nm using TEM measurements (Fig. 4(c)). This substantiates data from the zeta sizer as well.

#### 3.2.4. Fourier transform infrared spectroscopy (FT-IR)

**Fig. 5:**
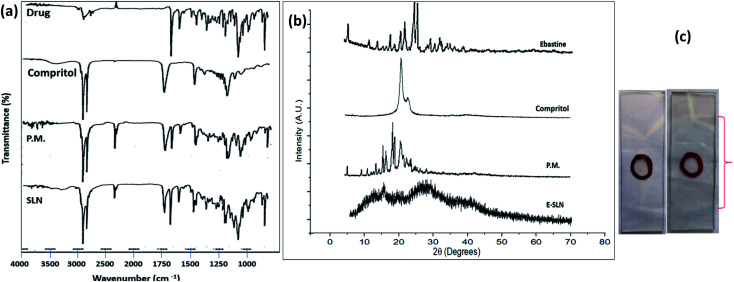
(a) FTIR spectra of ebastine, compritol, physical mixture and E-SLNs. (b) XRD patterns of ebastine, compritol, physical mixture and E-SLNs. (c) Spreadability of E-SLNs loaded hydrogel.

FTIR absorption spectrum of ebastine presented distinctive peaks at, 2971 cm^−1^ demonstrating C–H stretch of piperidine ring, 1700 cm^−1^ representing C=O stretch of ketone group that flanked piperidine group to left, 1104 cm^−1^ represent C–N stretch adopting a chair conformation, 1470 cm^−1^ represent C=C stretching due to vibration. Compritol showed absorption peaks at, 2917 cm^−1^ indicating aromatic C–H stretch, 2640 cm^−1^ representing alkyl C–H stretch, 1740 cm^−1^ shows C=O stretch due to presence of ketone group, 1480 cm^−1^ represent O–H bend, 1240 cm^−1^ represents C–H bands. The FTIR spectrum of E-SLNs presented in Fig. 5(a) showed all characteristic absorption peaks, indicating the absence of any physical or chemical interaction between ebastine and compritol.

#### 3.2.5. Powder X-ray diffractometry (PXRD)

XRD analysis of ebastine in Fig. 5(b) shows its crystalline structure with principal peaks between 10–35° degrees (2*θ*). A sharp crystalline peak of compritol was observed at 20° and 25° degrees with high intensity. However, these peaks were not present in the ebastine loaded solid lipid nanoparticles produced with compritol, indicating that the drug has been converted into amorphous form and is encapsulated into SLNs.

### 3.3. Characterization of E-SLNs loaded hydrogel

#### 3.3.1. Visual appearance, pH, and drug content

The prepared E-SLNs loaded chitosan hydrogel was transparent in appearance. The gel had no grittiness or lumps, indicating the homogeneity of gel. The average pH of E-SLNs loaded hydrogel was 7.61 ± 0.09, which indicates that it is suitable for topical application.

#### 3.3.2. Spreadability%

The spreadability of gel formulations also influences the therapeutic effectiveness of the gel. The mean spreadability of E-SLNs hydrogel was 283.3 ± 0.076%, indicating good spreadability (Fig. 5(c)).

#### 3.3.3. Drug content determination

Average drug content observed in E-SLNs loaded hydrogel was 93.8 ± 2.36%. It indicates that ebastine was evenly distributed in the hydrogel and drug loss was negligible during preparation of E-SLNs loaded hydrogel.

#### 3.3.4. Rheological measurements

**Fig. 6:**
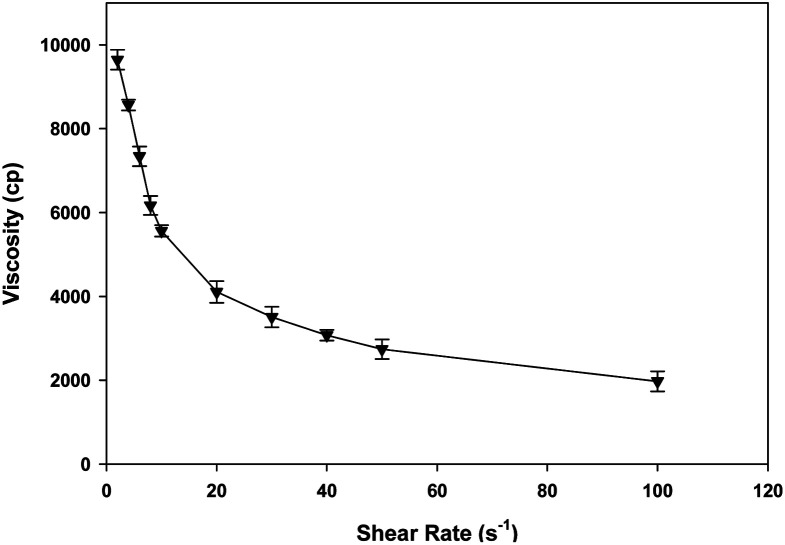
Rheological behaviour of E-SLNs loaded hydrogel. (*p* < 0.05).

E-SLNs loaded hydrogel viscosity varied inversely with shear rate. With increase in shear rate, the viscosity of gel decreased subsequently showing shear thinning or pseudoplastic behaviour (Fig. 6). This behaviour shows that E-SLNs loaded hydrogel is a non-Newtonian fluid.

#### 3.3.5. Swelling behaviour

**Fig. 7:**
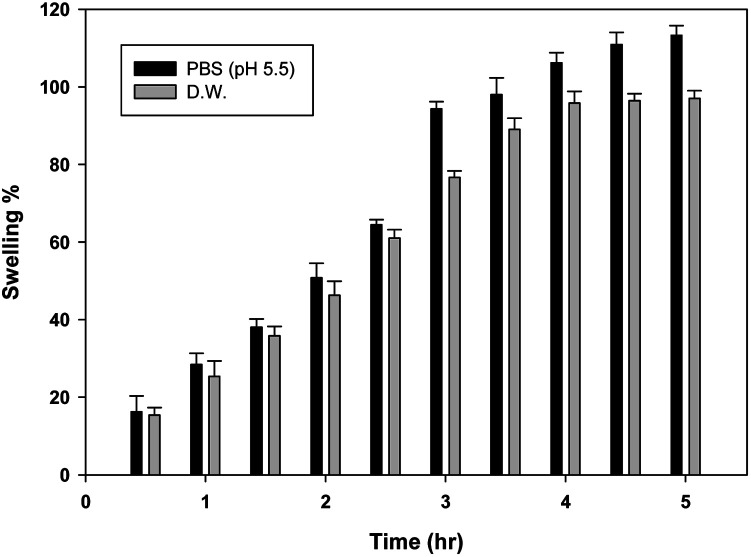
Swelling behaviour of E-SLNs loaded hydrogel in water and PBS (pH 5.5). (*p* < 0.05).

Swelling observed is less in water as compared to PBS (pH 5.5), because the swelling ratio of chitosan is always more in an acidic medium. Swelling% of E-SLNs loaded hydrogel in PBS 5.5 and water were 113.3% and 97.02% respectively (Fig. 7).

#### 3.3.6. Extrudability

For the application of E-SLNs loaded hydrogel on the skin, its extrudability is an important factor. The average extrudability of E-SLNs loaded hydrogel is 433 ± 15.275 g, which is within the acceptable range.

**Table 2:** Kinetic models applied on E-SLNs

Formulations	Zero order		First order		Kosmeyer–Peppas			Higuchi		Hixon Crowell	
	*R* ^2^	*k*0	*R* ^2^	*k*1	*R* ^2^	*n*	kP	*R* ^2^	kH	*R* ^2^	kHC
E-SLN dispersion	0.2086	4.590	0.8731	0.175	0.9691	0.347	29.615	0.8846	20.462	0.8115	0.048
E-SLN loaded hydrogel	0.4757	3.972	0.8726	0.105	0.9642	0.414	21.353	0.9431	17.290	0.8115	0.029

### 3.4. *In vitro* release studies

**Fig. 8:**
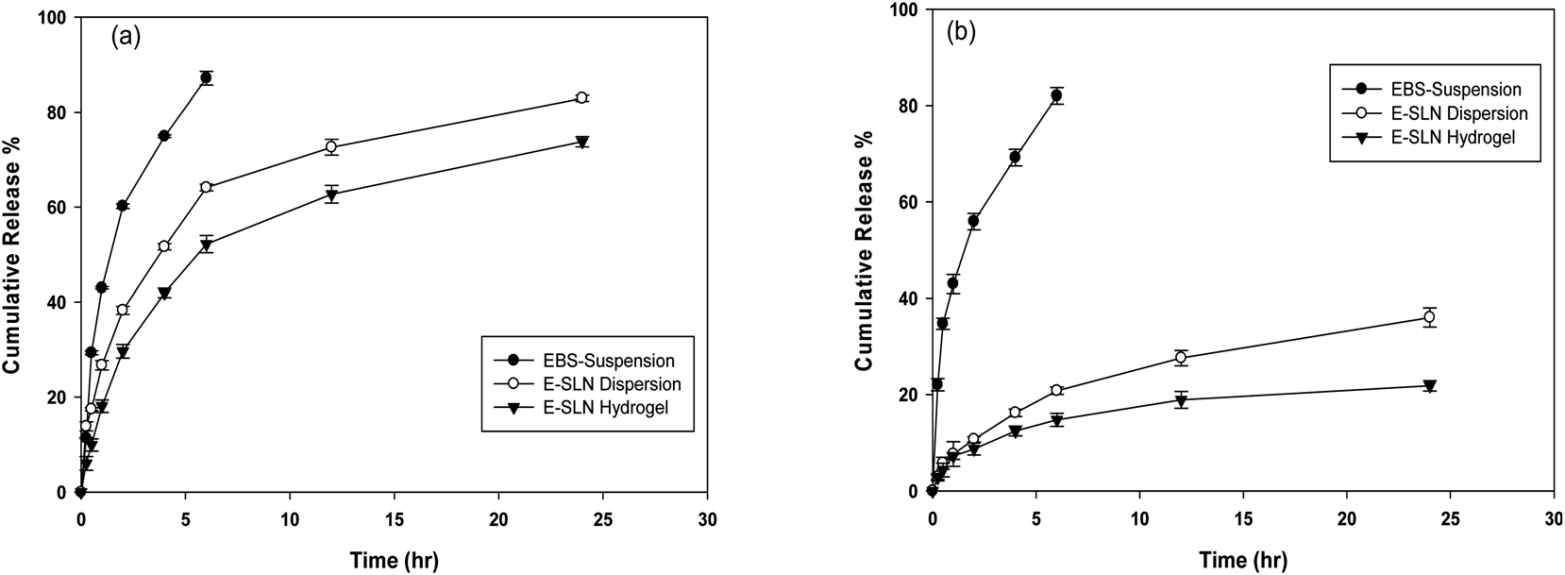
*In vitro* drug release profile for ebastine suspension, E-SLNs dispersion and E-SLNs loaded hydrogel (a) pH 5.5. (b) pH 7.4. (*p* < 0.05).

*In vitro* release investigation of ebastine suspension, E-SLNs, and E-SLNs loaded hydrogel was performed at pH 7.4 and pH 5.5. Ebastine suspension showed 81.9% release after the initial 6 hours, while E-SLNs showed 20% release in 6 hours and 36% release in 24 hours (Fig. 8(a)). E-SLNs loaded hydrogel showed 21% release after 24 hours at pH 7.4. At pH 5.5, Ebastine suspension showed 87% release after the initial 6 hours, while E-SLNs showed 64% release in 6 hours and 82.9% release in 24 hours. E-SLNs loaded hydrogel showed 73.7% release after 24 hours (Fig. 8(b)). Hydrogel showed more sustained release as compared to drug suspension and E-SLNs dispersion. Both E-SLNs and E-SLNs loaded hydrogel followed Korsmeyer–Peppas model. E-SLNs showed *R*^2^ equal to 0.9691 and “*n*” equal to 0.347. E-SLNs loaded hydrogel showed *R*^2^ equal to 0.9642 and “*n*” equal to 0.414 (Table 2). Value of *n* suggests that both formulations follow Fick's law and release drug through diffusion pattern. Initial burst release will provide fast onset of action on topical application whereas later sustained release of drug will provide a longer duration of action.

### 3.5. *Ex vivo* permeation studies

**Fig. 9:**
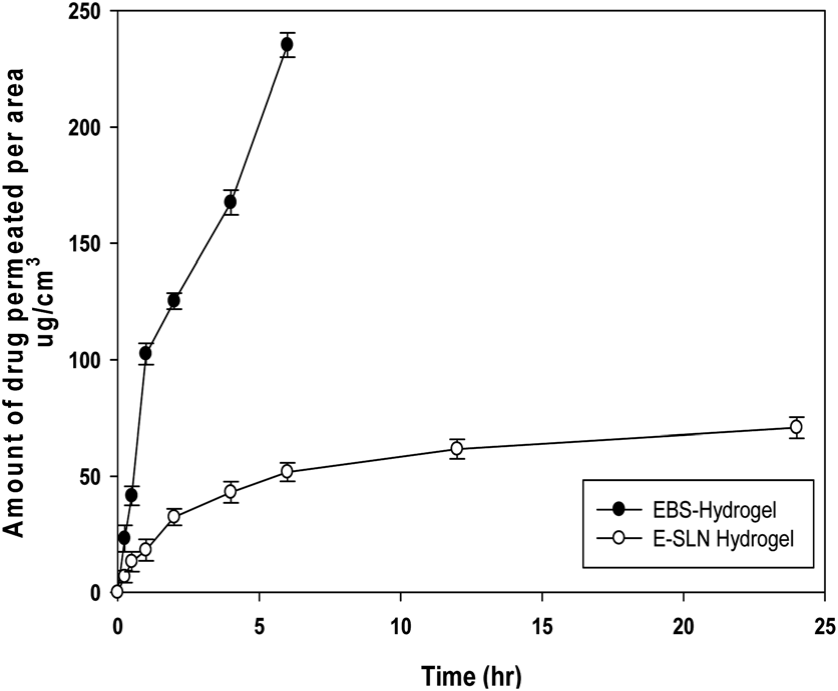
*Ex vivo* permeation of ebastine hydrogel and ESLNs loaded hydrogel. (*p* < 0.05).

The amount of ebastine permeated per area was calculated. Ebastine gel showed maximum permeation as compared to E-SLNs loaded hydrogel. 232.15 μg cm^−2^ of ebastine from ebastine hydrogel permeated through mice skin in 6 hours. While negligible amount of ebastine permeated in 24 hours through E-SLNs loaded hydrogel into receiver compartment *i.e.*, 70.76 μg cm^−2^. Results shown in Fig. 9 proves that the drug will provide topical effect on application.

### 3.6. Stability studies of E-SLNs loaded hydrogel

E-SLNs loaded hydrogel stored at temperatures 4 °C and 25–35 °C showed no significant change in pH and drug content for 6 months. Colour change was observed after 6 months at room temperature (Table 3).

**Table 3:** Stability studies of E-SLNs loaded hydrogel. (*p* < 0.05)

Physical appearance						
Temperature	Time	Colour change	Phase separation	Grittiness	Drug content%	pH
	0 Day	No	No	No	93.78 ± 2.36	7.61 ± 0.9
25–35 °C	30 Days	No	No	No	93.04 ± 3.41	7.58 ± 0.21
	90 Days	No	No	No	91.56 ± 1.61	7.44 ± 0.13
	180 Days	Yes	No	No	90.74 ± 4.07	7.38 ± 0.6
	0 Day	No	No	No	93.78 ± 2.36	7.61 ± 1
	30 Days	No	No	No	93.1 ± 0.03	7.61 ± 0.64
4 °C	90 Days	No	No	No	92.29 ± 1.23	7.59 ± 0.2
	180 Days	No	No	No	91.41 ± 1.42	7.55 ± 0.17

### 3.7. Skin irritation studies

**Fig. 10:**
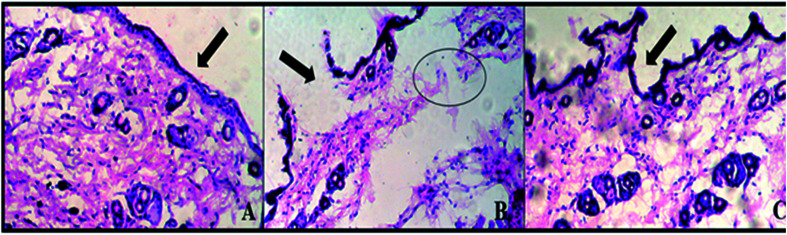
Histopathological evaluation of skin irritation (a) negative control (b) positive control (c) E-SLNs loaded hydrogel.

Draize scoring was used to evaluate skin irritation in mice. E-SLNs loaded hydrogel showed PDII of 0.33 which represents negligible irritation, but formalin-treated mice showed PDII of 3.33. Results showed that hydrogel caused slight irritation, while formalin caused moderate irritation (Table 4). Histopathological examination was performed to confirm the findings. Histopathological images indicate that skin tissues of the positive control group are damaged by formalin, but no tissue damaged occurred in the mice group treated with E-SLNs loaded hydrogel (Fig. 10).

**Table 4:** Skin irritation studies of E-SLNs using Draize scoring

Groups	Erythema score			Edema score			P.D.I.			PDII
	1 h	12 h	24 h	1 h	12 h	24 h	1 h	12 h	24 h	
Negative control	0	0	0	0	0	0	0	0	0	0
Positive control	1	3	3	1	1	2	1	4	5	3.33
E-SLN loaded hydrogel	0	1	0	0	0	0	0	1	0	0.33

### 3.8. *In vivo* allergic contact dermatitis studies

#### 3.8.1. Ear swelling response

**Fig. 11:**
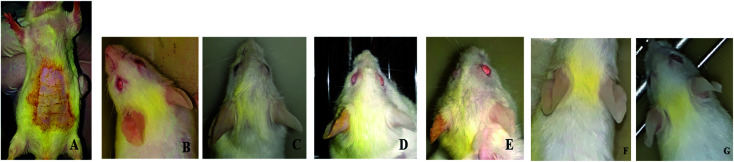
*In vivo* ACD Studies, (a) abdominal sensitization. (b) After 1st ear sensitization. (c) After 2nd ear sensitization. (d) After 3rd ear sensitization. (e) After 4th sensitization. (f) 5 days after application of E-SLNs loaded hydrogel. (g) 15 days after application of E-SLNs loaded hydrogel.

**Fig. 12:**
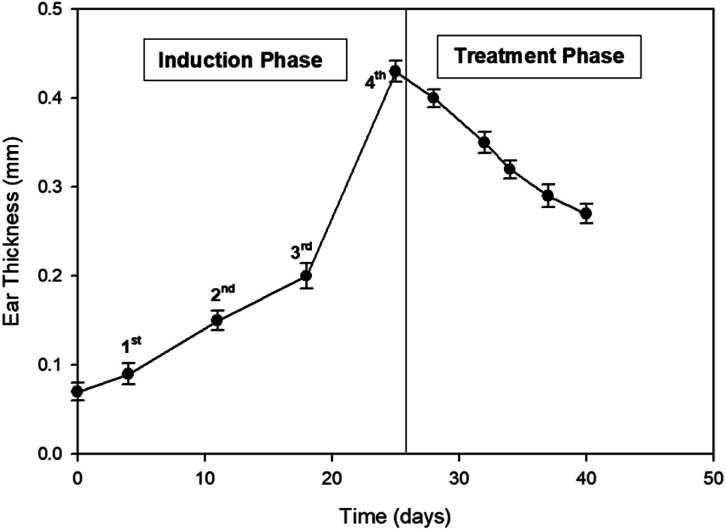
Ear swelling response of mice on ACD induction and alleviation. (*p* < 0.05).

During the induction phase, initially swelling increased slowly but peaked after 3^rd^ application (Fig. 11). Normal ear thickness was observed to be 0.07 mm at first, which increased to 0.43 mm after several sensitizations. Swelling of ear increased to maximum after the 4^th^ application. In the treatment phase, swelling and redness due to inflammation slowly decreased after successive application of E-SLNs loaded hydrogel. Ear thickness was decreased from 0.43 mm to 0.27 mm after 15 days treatment (Fig. 12). This swelling response showed the anti-allergic property of E-SLNs loaded hydrogel.

#### 3.8.2. Histopathological studies

**Fig. 13:**
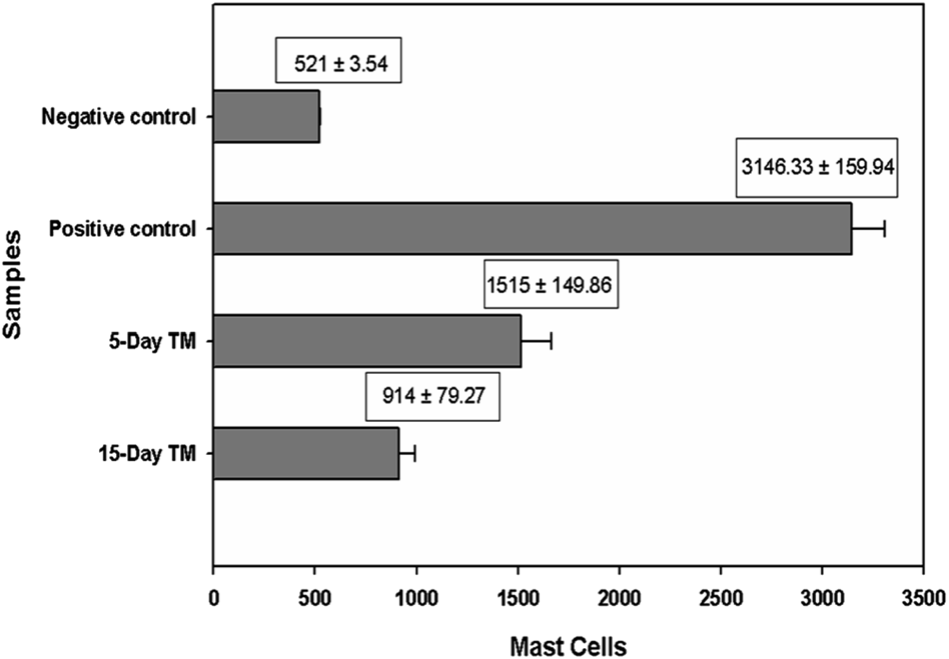
Mast cells count in dermis of mice skin. (*p* < 0.05).

**Fig. 14:**
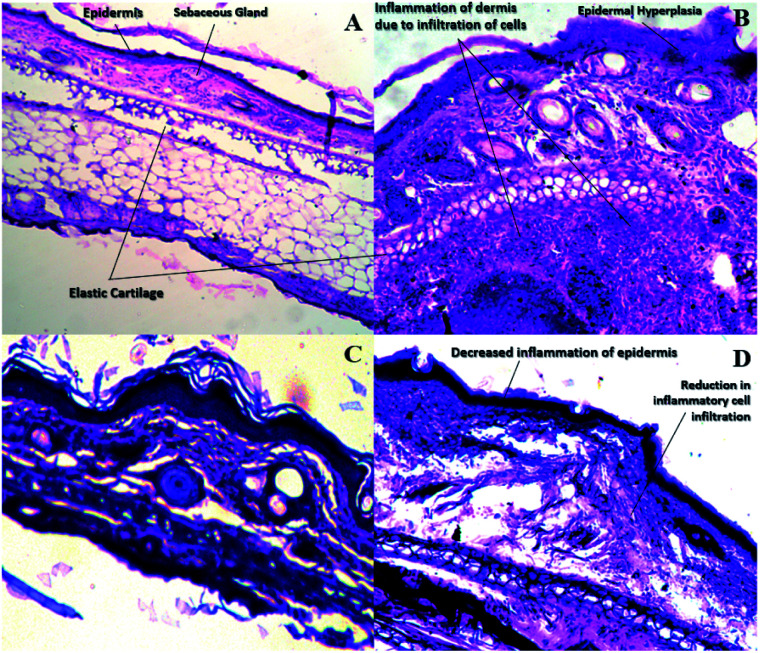
Histopathological findings of mice ear stained with H and E. (a) negative control. (b) positive control (c) 5-days treatment with E-SLNs loaded hydrogel. (d) 15- control. (b) Positive control (c) 5-days treatment with E-SLNs loaded hydrogel (d) 15-days treatment with E-SLNs loaded hydrogel.

**Fig. 15:**
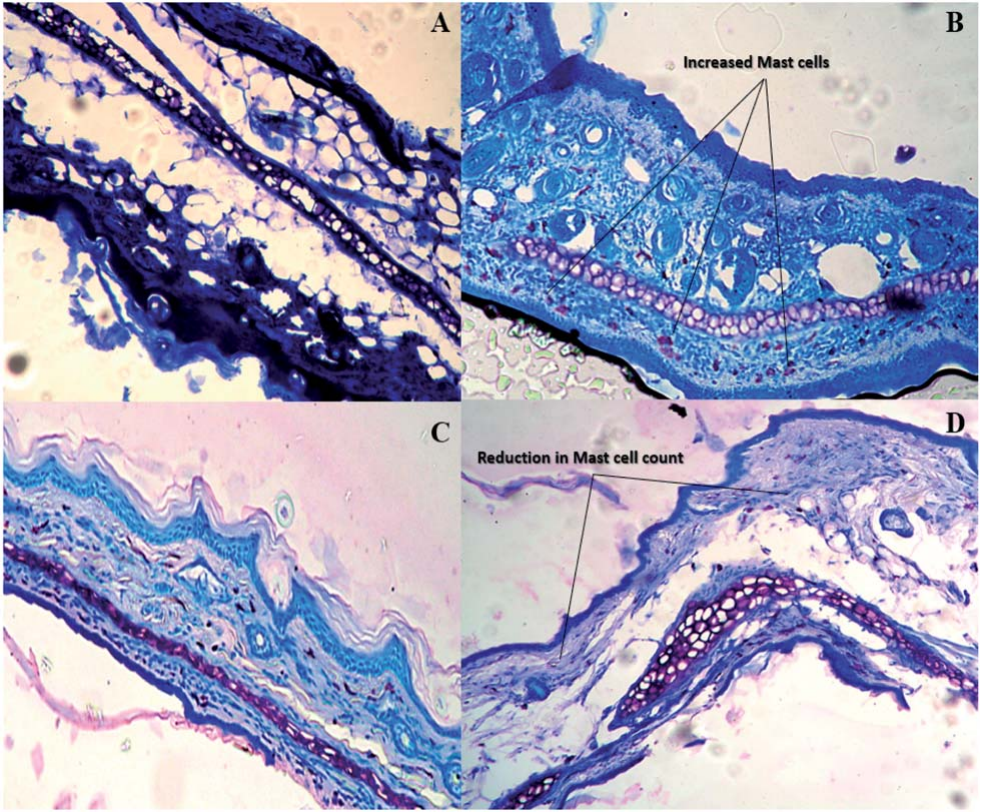
Histopathological findings of mice ear stained with toluidine blue. (a) Negative control. (b) Positive control (c) 5 days treatment with E-SLNs loaded hydrogel. (d) 15-control. (b) Positive control (c) 5 days treatment with E-SLNs loaded hydrogel (d) 15 days treatment with E-SLNs loaded hydrogel.

After induction, the ear dermis of mice with contact dermatitis shows severe edema and prominent fibroplasia as compared to the negative control. This infiltration decreased after 5 days of treatment of E-SLNs loaded hydrogel and decreased further, after 15 days of treatment. The thickness of the epidermis is increased in positive control when compared with negative control. Thickness decreased after successive application of E-SLNs loaded hydrogel (Fig. 14). Mast cells count is increased in the superficial dermis of ACD induced mice ear from 521 to 3146. Mast cells count was later reduced by E-SLNs loaded hydrogel application for 5 days and 15 days to 1515 and 914 respectively, as shown in Fig. 13. The visibility of mast cells was increased in toluidine blue staining (Fig. 15). Changes in the histopathological structure were negligible in the left ear applied with vehicle only.

## 4. Discussion

Ebastine has approximate bioavailability of 1%, which indicates that it has low solubility. For ebastine to be more effective, its bioavailability must be increased, which could be achieved by increasing its solubility. Compritol and tween-80 were chosen for the preparation of ebastine nanoparticles. Hot homogenization and cold dilution method were used for the preparation of E-SLNs. The central composite design was selected to optimize E-SLNs using design expert version 11. Ten formulations were proposed with two centre points, each run had a varying concentration of drug and lipid while keeping the concentration of surfactant constant. The effect of change in concentration of each factor was observed in terms of P.S., zeta potential, PDI, and entrapment efficiency. Formulation having minimum particle size with maximum zeta potential and EE% were selected through software. Tween 80 has long-chain fatty acid (oleic acid), which increases its hydrophobicity. Hydrophobic chains arrange more tightly in the oil phase when dispersed in cold water which reduces particle size. As the melting point of ebastine is higher than solid lipid, an increase in the ebastine concentration causes an increase in the particle size of SLNs because the viscosity of melted lipid increases at high drug concentration.^42^ Zeta potential between ±25 mV indicates the stability of nanoparticles. An increase in drug and solid lipid concentration increases zeta potential value when the concentration of surfactant is kept constant. This is mainly due to the presence of oxygen species on the nanoparticle surface, which provides a negative charge.^43^ EE% increased with an increase in the concentration of drug and lipid. Increased lipid content allows for additional area to encapsulate more drug, lowering its partition in the outer phase. This could also be attributed to an increase in medium viscosity, which results in faster solidification of nanoparticles.^44^ TKF 10 formulation was chosen, as it showed optimum results in every perspective. For topical application, the hydrogel was prepared using chitosan for the controlled release of the drug. The biocompatibility and biodegradability of chitosan have made it a desirable material for topical applications.^45^ Chitosan was selected due to its anti-inflammatory properties, which will aid in treating allergic contact dermatitis. High molecular weight chitosan has higher anti-inflammatory properties. It induces anti-inflammatory cytokine IL-10 production in animals.^46^ Swelling ratio is higher at pH 5.5 as compared to water because chitosan has high swelling at acidic pH. When the pH value of the medium is increased further, the swelling of chitosan hydrogels decreases. Because the concentration of H+ was high under low pH conditions, so chitosan is more likely to undergo protonation of the amine group to produce –NH_3_^+^. Amine group deprotonates when pH increases, this decreases repulsion between hydrogel polymer chains.^47^ On performing rheological studies, the viscosity of E-SLNs loaded hydrogel decreased on increasing the shear rate indicating that it is a non-Newtonian fluid with shear-thinning or pseudoplastic behaviour, which is a feature of high molecular weight polysaccharide solutions.^48^ Release of ebastine from E-SLNs and E-SLNs loaded hydrogel was evaluated at two different pH *i.e.*, 5.5 and 7.4 for comparison. The slow release of ebastine from SLNs as compared to drug suspension indicates that the drug is entrapped and dispersed uniformly throughout the system. Tween 80 reduces interfacial tension between dissolution medium the SLNs, this decreases aggregation of drug particles and improves drug dissolution rate. The long lipophilic fatty acid chain of compritol also prolongs the release of ebastine.^49^ Kinetic models were applied to investigate the mechanism and pattern of release. Korsmeyer–Peppas was the best-fitted model with a value of *n* equal to 0.347, indicating release of drug according to Fick's law. Drug released through diffusion from homogeneous matrix system of lipid.^50^ Histopathological studies were performed to analyse the changes in tissue structure of the ear dermis. Ear dermis of mice after induction of contact dermatitis shows severe edema, it started to develop after picryl chloride application. Prominent fibroplasia is seen as compared to the normal ear. Inflammatory cell infiltration can be seen between the dermis and elastic cartilage. Eosinophils, neutrophils, and mononuclear cells were the common infiltrated cells present. This infiltration decreased after 5 days of treatment of E-SLNs loaded hydrogel and decreased further, after 15 days of treatment. The thickness of the epidermis is increased due to hyperplasia of keratinocytes and hyperkeratosis in positive control when compared with negative, which afterward decreased on treatment with E-SLNs loaded hydrogel.

## 5. Conclusions

Ebastine-loaded SLNs were formulated and optimized, using central composite design and response surface methodology. The final formulation particle size was within nano-size and PDI was less than 0.5. Physically, crystalline ebastine was converted into an amorphous form when fabricated into SLNs. E-SLNs were successfully loaded into chitosan hydrogel with suitable pH, spreadability, homogeneity, and swelling behaviour. Ear swelling behaviour, histopathological studies, and mast cell count confirmed that E-SLNs loaded hydrogel alleviated the symptoms of allergic contact dermatitis in the animal model after successive applications.

## Author contributions

We affirm that this work was completed by the authors mentioned in this article and that the authors will bear all liabilities relating to aspects relevant to the content of the article.

## Conflicts of interest

There are no conflicts to declare.

## Supplementary Material

RA-011-D1RA06283B-s001

RA-011-D1RA06283B-s002

RA-011-D1RA06283B-s003

RA-011-D1RA06283B-s004

RA-011-D1RA06283B-s005

RA-011-D1RA06283B-s006

RA-011-D1RA06283B-s007

RA-011-D1RA06283B-s008

RA-011-D1RA06283B-s009

RA-011-D1RA06283B-s010
