# Supplementary figures and images for: Chitosan hydrogel for topical delivery of ebastine loaded solid lipid nanoparticles for alleviation of allergic contact dermatitis
Source: RSC Adv. 2021 Nov 22;11(59):37413–25. doi: 10.1039/d1ra06283b (PMC9043795; doi:10.1039/d1ra06283b)

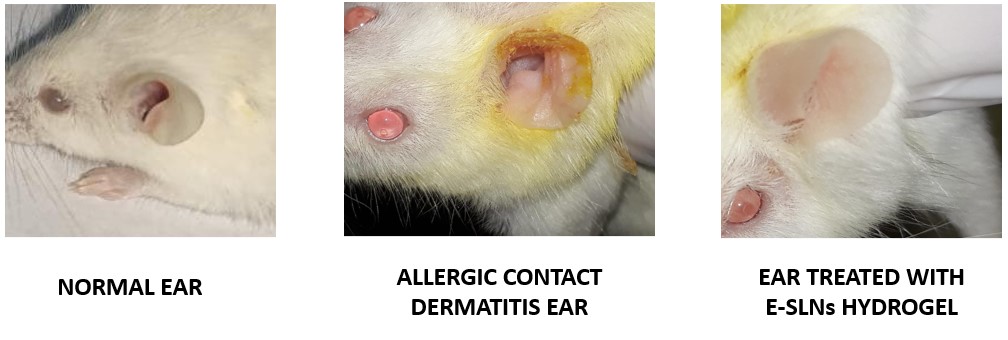

Supplement: RA-011-D1RA06283B-s004 [file RA-011-D1RA06283B-s004.jpg]

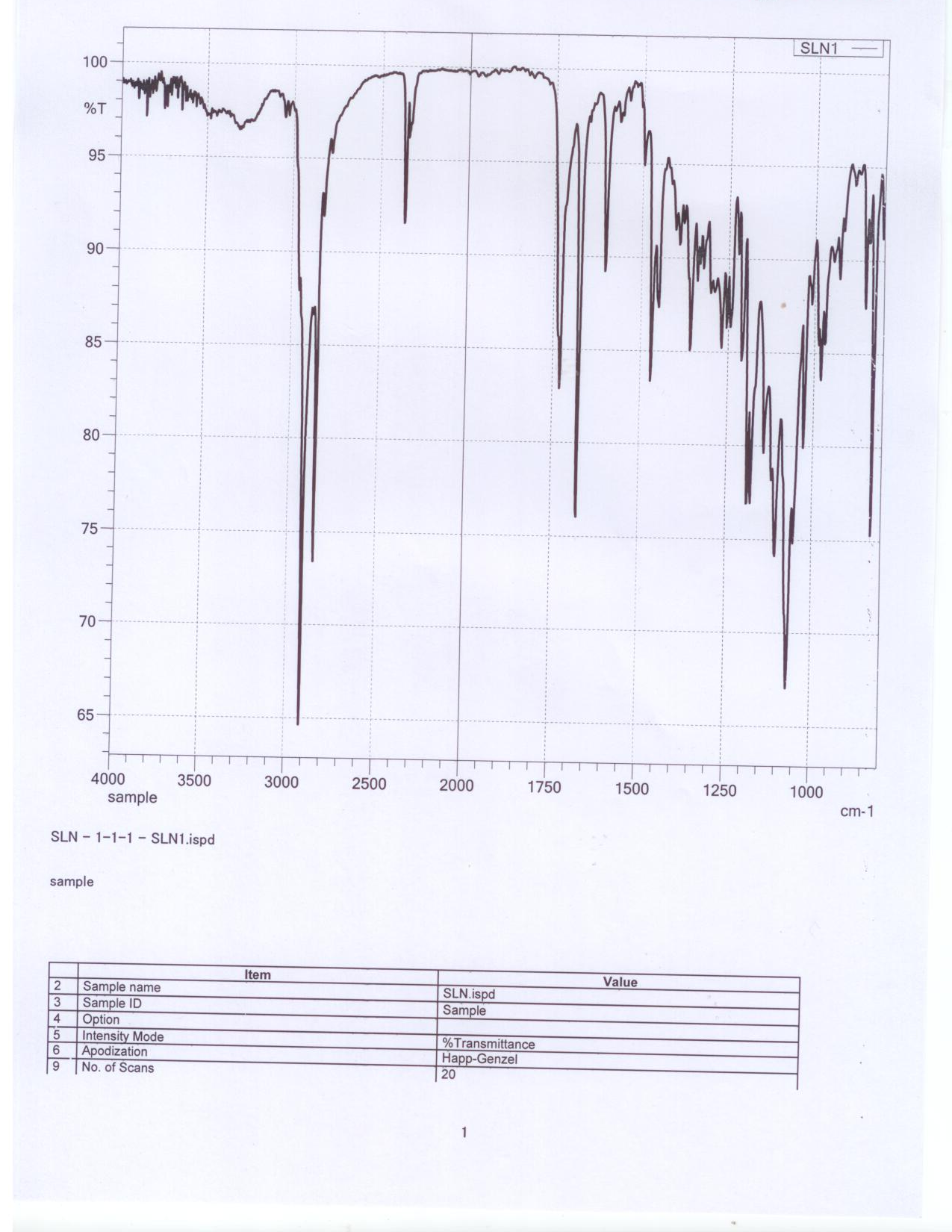

Supplement: RA-011-D1RA06283B-s007 [file RA-011-D1RA06283B-s007.tif]

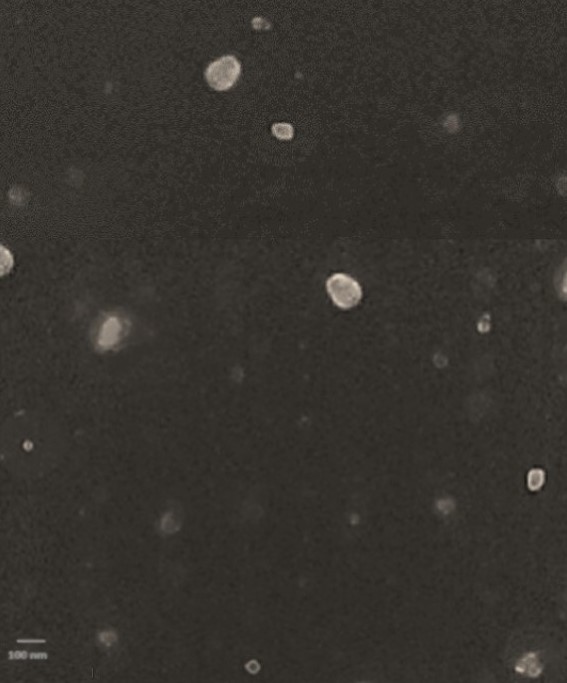

Supplement: RA-011-D1RA06283B-s008 [file RA-011-D1RA06283B-s008.jpg]

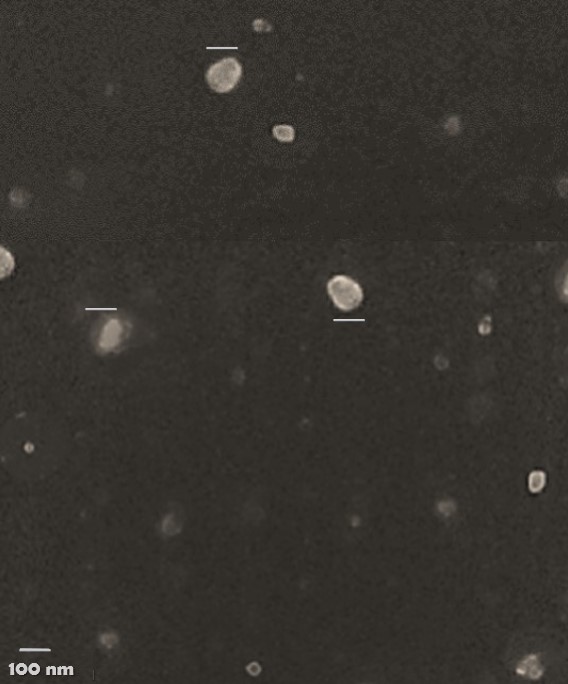

Supplement: RA-011-D1RA06283B-s009 [file RA-011-D1RA06283B-s009.jpg]

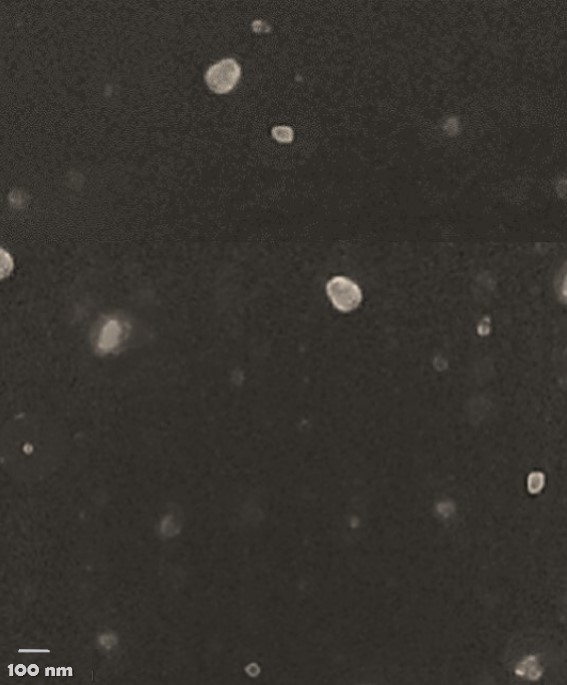

Supplement: RA-011-D1RA06283B-s010 [file RA-011-D1RA06283B-s010.jpg]
